# Functions and mechanisms of A-to-I RNA editing in filamentous ascomycetes

**DOI:** 10.1371/journal.ppat.1012238

**Published:** 2024-06-06

**Authors:** Zeyi Wang, Zhuyun Bian, Diwen Wang, JinRong Xu

**Affiliations:** Department of Botany and Plant Pathology, Purdue University, West Lafayette, Indiana, United States of America; Rutgers University, UNITED STATES

## Abstract

Although lack of ADAR (adenosine deaminase acting on RNA) orthologs, genome-wide A-to-I editing occurs specifically during sexual reproduction in a number of filamentous ascomycetes, including *Fusarium graminearum* and *Neurospora crassa*. Unlike ADAR-mediated editing in animals, fungal A-to-I editing has a strong preference for hairpin loops and U at −1 position, which leads to frequent editing of UAG and UAA stop codons. Majority of RNA editing events in fungi are in the coding region and cause amino acid changes. Some of these editing events have been experimentally characterized for providing heterozygote and adaptive advantages in *F*. *graminearum*. Recent studies showed that FgTad2 and FgTad3, 2 ADAT (adenosine deaminase acting on tRNA) enzymes that normally catalyze the editing of A34 in the anticodon of tRNA during vegetative growth mediate A-to-I mRNA editing during sexual reproduction. Stage specificity of RNA editing is conferred by stage-specific expression of short transcript isoforms of *FgTAD2* and *FgTAD3* as well as cofactors such as *AME1* and *FIP5* that facilitate the editing of mRNA in perithecia. Taken together, fungal A-to-I RNA editing during sexual reproduction is catalyzed by ADATs and it has the same sequence and structural preferences with editing of A34 in tRNA.

A-to-I RNA editing catalyzed by ADAR (adenosine deaminase acting on RNA) enzymes that converts adenosines (As) to inosines (Is) by deamination is the most common form of RNA editing in metazoans. Because inosines have similar properties with guanosines, ADAR-mediated editing can alter codons and affect RNA folding, splicing, and interactions [1,2]. ADARs typically contain a deaminase domain and 1–3 double-stranded RNA binding domains (dsRBDs), and RNA editing occurs throughout the body. Because ADARs are unique to metazoans, A-to-I editing was considered to occur only in animals. To date, RNA editing has not been reported in nuclear-encoded RNA in plants. However, although lack of ADAR homologs in the genome, genome-wide A-to-I RNA editing has been reported in several filamentous ascomycetes during sexual reproduction, including *Fusarium graminearum*, *F*. *verticillioides*, *Neurospora crassa*, *Sordaria macrospora*, and *Pyronema confluens* [3–6]. This review will summarize unique features, distinct functions, and underlying mechanisms of stage-specific A-to-I RNA editing in fungi.

## Genome-wide RNA editing during sexual reproduction in filamentous ascomycetes

Fungal A-to-I RNA editing was first reported in *F*. *graminearum*, a homothallic Sordariomycetes that forms asci in perithecia and causes Fusarium head blight (FHB) of wheat and barley. Because ascospores are the primary inoculum, sexual reproduction is a critical step in its infection cycle. To date, over hundreds of genes important for sexual development have been identified in *F*. *graminearum* [7,8]. One of them is the *PUK1* protein kinase gene that is required for normal ascosporogenesis and ascospore release [7]. Interestingly, the coding region of *PUK1* has 2 tandem stop codons TAGTAG (1830–1835) that are converted to TGGTGG in its cDNA fragments amplified by reverse transcription PCR (RT-PCR), indicating the occurrence of A-to-I editing [3]. Stranded RNA-seq analysis with vegetative hyphae, conidia, and perithecia collected at 8 days postfertilization (dpf) showed that genome-wide A-to-I editing specifically occurs during sexual reproduction. Similar to A-to-I editing in humans, the average editing level is 14.8% for the 26,056 editing events identified in 8-dpf perithecia, although A^1831^ and A^1834^ of *PUK1* were edited at 91% and 99%, respectively, in *F*. *graminearum* [3].

Genome-wide RNA editing during sexual reproduction has also been reported in several other filamentous ascomycetes. In *N*. *crassa*, over 41,000 RNA editing sites were identified by RNA-seq analysis with perithecia collected at 3 to 6 dpf, with some of them being unique to specific stages [4]. In *F*. *graminearum*, a time-course RNA-seq analysis with mating cultures at 3 to 8 dpf also lead to the identification of over 40,000 editing sites, including editing events that were present only in early or late sexual developmental stages [9]. Comparative analysis showed that some of these RNA editing events occur at the same sites in their orthologs in *N*. *crassa*, *F*. *graminearum*, and *N*. *tetrasperma*. One of these conserved editing sites is A^1831^ of *PUK1*. A-to-I RNA editing also occurs specifically during sexual reproduction in *S*. *macrospora*, another model Sordariomycetes [5]. Although RNA editing events were detected in protoperithecia, young perithecia had significant more editing sites. No RNA editing was observed in the *pro1* and *nxo1* mutants of *S*. *macrospora* that are blocked in perithecium development [5].

Putative RNA-editing events have been reported in several Basidiomycetes, including *Ganoderma lucidum* [10], *Fomitopsis pinicol*, and *Polyporales* species. However, a more careful analysis showed that these fungi do not have A-to-I RNA editing and erroneously identified A-to-G variants are caused by sequencing errors and/or problems with genome assemblies [11]. The budding and fission yeasts that form naked asci and belong to Saccharomycotina and Taphrinomycotina, respectively, also lack mRNA editing. Thus, genome-wide A-to-I editing during sexual reproduction appears to occur only in Pezizomycotina. Whereas all other species with reported A-to-I editing are Sordariomycetes, *P*. *confluens* is a Pezizomycetes in which 2,772 sexual-specific editing sites have been identified, including 15 of them that were experimentally confirmed [5], indicating that stage-specific RNA editing also occurs in Pezizomycetes during sexual reproduction, although it may be less abundant as in Sordariomycetes. However, genome-wide RNA editing was not identified in cleistothecia of *Aspergillus nidulans*, a Eurotiomycetes, and apothecia of *Botrytis cinerea*, a Leotiomycetes [12]. Therefore, it remains to be determined which classes or orders in Pezizomycotina have RNA editing.

## Majority of fungal RNA editing sites are in the coding region and cause amino acid changes

In metazoans, the vast majority of editing events occurs outside the coding region (CDS). Even for editing sites in the CDS, most of them do not cause amino acid changes (synonymous editing). Among over 3 million editing sites identified in humans, only 1,741 of them (<0.06%) are in the CDS and 552 of them (<0.02%) cause amino acid changes [13]. In *F*. *graminearum*, 70% of the editing sites are in the CDS and 64% of them are nonsynonymous or missense editing. In *N*. *crassa*, 64% of the editing sites are in the CDS and 52% of them are nonsynonymous.

With majority of editing events being nonsynonymous in fungi, A-to-I RNA editing significantly increases the proteome complexity during sexual reproduction [3–5]. In addition, because the average editing level is approximately 15%, RNA editing at a specific site will lead to the coexisten2 two genes important but not essential for ascosporogenesis that share conserved missense editing sites with their orthologs in *N*. *crassa*. For *CME11*, coexpression of unedited and edited versions is necessary for the complementation of the defect of the *cme11* deletion mutant in ascosporogenesis [14], which provides the first experimental evidence for the heterozygote advantage of nonsynonymous RNA editing.

## Sequence and structure preference of fungal RNA editing

**Fig 1:**
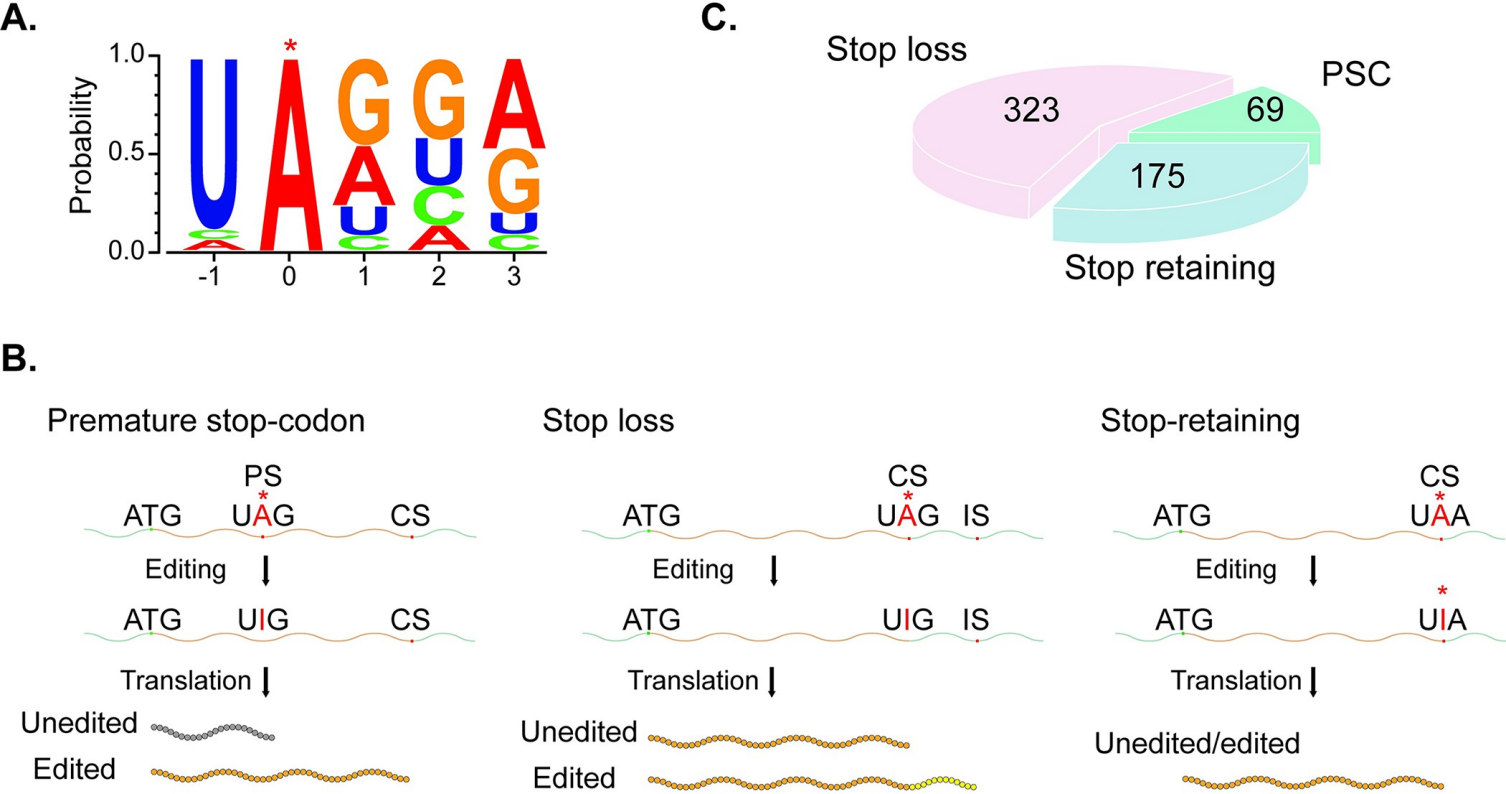
Sequence preference of mRNA editing and stop codon editing in *F*. *graminearum* editing in fungi. **(A)** A-to-I mRNA strongly favors U at the −1 position. **(B)** A-to-I editing of UAG leads to premature stop codon correction (PSC) and stop-loss editing. Editing of UAA to UGA retains the stop codon. PS, CS, and IS represent premature, canonical, and in-frame stop codons, respectively. **(C)** PSC, stop-loss, and stop-retaining editing events in *F*. *graminearum*.

When the nucleotide sequences flanking the edited adenosines were compared, over 70% of the editing sites have U at the −1 position in *F*. *graminearum* (Fig 1A). Similar strong preference for U at −1 position was observed in *N*. *crassa*. Furthermore, it appears that A-to-I RNA editing favors A or G at +1 and +3 positions (Fig 1A) in *F*. *graminearum* [15]. In addition, adenosines in the preferred flanking sequences (Fig 1A) tend to have higher editing levels than those in nonpreferred flanking sequences, suggesting that nucleotides surrounding the editing site influence both the specificity and efficiency of A-to-I editing in fungi. In contrast, ADAR-mediated RNA editing in animals has only a weak preference for the flanking sequences, with the depletion of G at the −1 and slight enrichment of G at the +1 [13].

RNA editing in fungi also differs from metazoans in the preference of predicted secondary structures of edited mRNA. Whereas ADAR-mediated editing events preferentially occur in the stem region in metazoans, RNA editing in fungi displays a preference for adenosines on hairpin loops of predicted secondary mRNA structures [3,4]. Furthermore, edited adenosines in the loop region tend to have higher editing levels than those in the stem region in *F*. *graminearum* and *N*. *crassa*, indicating that secondary structures of mRNA also influence the occurrence of A-to-I editing in fungi [3,15].

## Editing of stop codons during fungal sexual development

Because of the strong preference for U at −1 position, unlike ADAR-mediated mRNA editing, RNA editing at stop codons is relatively frequent in fungi. Similar to the tandem stop codons in *PUK1*, another 70 genes in *F*. *graminearum* have stop codons in the CDS and require PSC (premature stop correction) editing of UAG to UGG to synthesize full-length functional proteins. A number of these genes, including *AMD1*, *FgRID*, *FgAMA1*, and *FgBUD14*, have been individually characterized for their functions in ascus development and ascospore formation or release [3,16–18]. Whereas FgAma1 is an activator of the meiotic anaphase promoting complex, FgRid is a DNA methyltransferase orthologous to *N*. *crassa* Rid that is essential for repeat-induced point mutation during sexual reproduction [17,18]. *AMD1* encodes a major facilitator superfamily (MFS) domain protein that is important for ascus maturation and ascospore discharge [16]. For *FgBUD14*, both editing of UA^1334^G to UGG and alternative splicing of intron 2 containing this PSC site occur during sexual reproduction. Recently, 16 other genes with PSC editing in *F*. *graminearum* were found to be important for fruiting body development [19]. For some of them, restorative editing of the PSC sites appears to have adaptive advantages for balancing the survival-reproduction trade-offs. In *F*. *graminearum* and other plant pathogens, sexual fruiting bodies are important for overwintering and ascospores function as the primary inoculum. PSC editing events in genes essential for ascospore development and releasing indicate the importance of RNA editing in the infection cycle of these plant pathogenic fungi.

Besides PSC editing, editing of the canonical stop codon UAG to UGG (stop-loss editing) will result in a C-terminal extension in proteins translated from edited transcripts (Fig 1B), although ribosome stalking may affect translation efficiency and mRNA stability. Both *F*. *graminearum* and *N*. *crassa* have over hundreds of genes with stop-loss editing events. Unfortunately, unlike extensive characterization of the importance of PSC editing during sexual reproduction [3,16–19], there is no report on the function of stop-loss editing events in sexual reproduction. Another type of editing events at canonical stop codons is stop-retaining editing of UAA to UGA. Although none of the stop-retaining editing events have been functionally characterized, it is likely that they are not as important as PSC or stop-loss editing events. In *F*. *graminearum*, there are significantly more stop-loss editing events than stop-retaining editing events (Fig 1C), which provides indirect evidence on the importance of stop-loss editing during fungal sexual reproduction.

## ADATs are responsible for mRNA editing during sexual reproduction

Although it lacks ADAR homologs, *F*. *graminearum* has genes that encode proteins with adenosine or cytosine deaminase domains. Whereas 2 of them are essential genes orthologous to yeast *TAD2* and *TAD3*, mutants deleted of the other 16 individually have no obvious defects in A-to-I mRNA editing [20]. In *S*. *cerevisiae*, *TAD2* and *TAD3* are 2 ADAT (adenosine deaminase acting on tRNA) genes. The Tad2 and Tad3 ADATs form a heterodimer to catalyze the editing of A34 on the anticodon loop of tRNA [21], which is similar to the structural preference of loops for RNA editing in fungi. Editing of A34 in tRNA by ADATs also shares similar preference for flanking nucleotide sequences with fungal RNA editing, including 100% U at −1 position. Interestingly, both *FgTAD2* and *FgTAD3* have 2 transcript isoforms. Whereas the longer isoforms are expressed in both vegetative hyphae and perithecia, the shorter isoforms are specifically expressed during sexual reproduction and their expression levels increase proportionally with the increase of editing events in developing perithecia from 3 to 8 dpf [22,23]. In fact, the shorter isoforms of *FgTAD2* and *FgTAD3* become more abundant than the longer ones after 4 dpf. These observations implicate the involvement of Tad2 and Tad3 orthologs in A-to-I mRNA editing during sexual reproduction in fungi.

To generate mutant alleles of *FgTAD2* with the repeat-induced point (RIP) mutation approach [24], ascospore progeny were isolated by single-spore isolation from a transformant of the wild type carrying an ectopically integrated, nonfunctional *FgTAD2* fragment [23]. Because of the importance of tRNA editing for hyphal growth and PSC editing for ascosporogenesis [3,16–18], ascospore progeny that were normal in vegetative growth (likely no defects in editing of A34 in tRNA) and but defective in ascosporogenesis (mRNA editing) were identified and sequenced for mutations in *FgTAD2*. Among the 17 RIP mutations identified in 9 such ascospore progeny, only the H352Y and Q375*(nonsense) mutations were present in 2 or more progeny [23]. The *FgTAD2*^H352Y^ and *FgTAD2*^Q375^* mutants generated by in situ knock-in and knock-out had similar defects with the original RIP progeny in ascosporogenesis but were normal in vegetative growth. RNA-seq analysis showed that the H352Y and Q375* mutants, in particular the latter, was significantly reduced in editing events and editing levels. In an in vitro editing assay with *PUK1* mRNA, FgTad2-His proteins affinity-purified from perithecia, but not from vegetative hyphae, had mRNA editing activities. Moreover, the H352Y mutation was demonstrated to affect the editing of *PUK1* mRNA by FgTad2^H532Y^ but not its ability to edit tRNA [23]. Results from these genetic analyses and biochemical assays indicate that *FgTAD2* is responsible for A-to-I mRNA editing during sexual reproduction.

**Fig 2:**
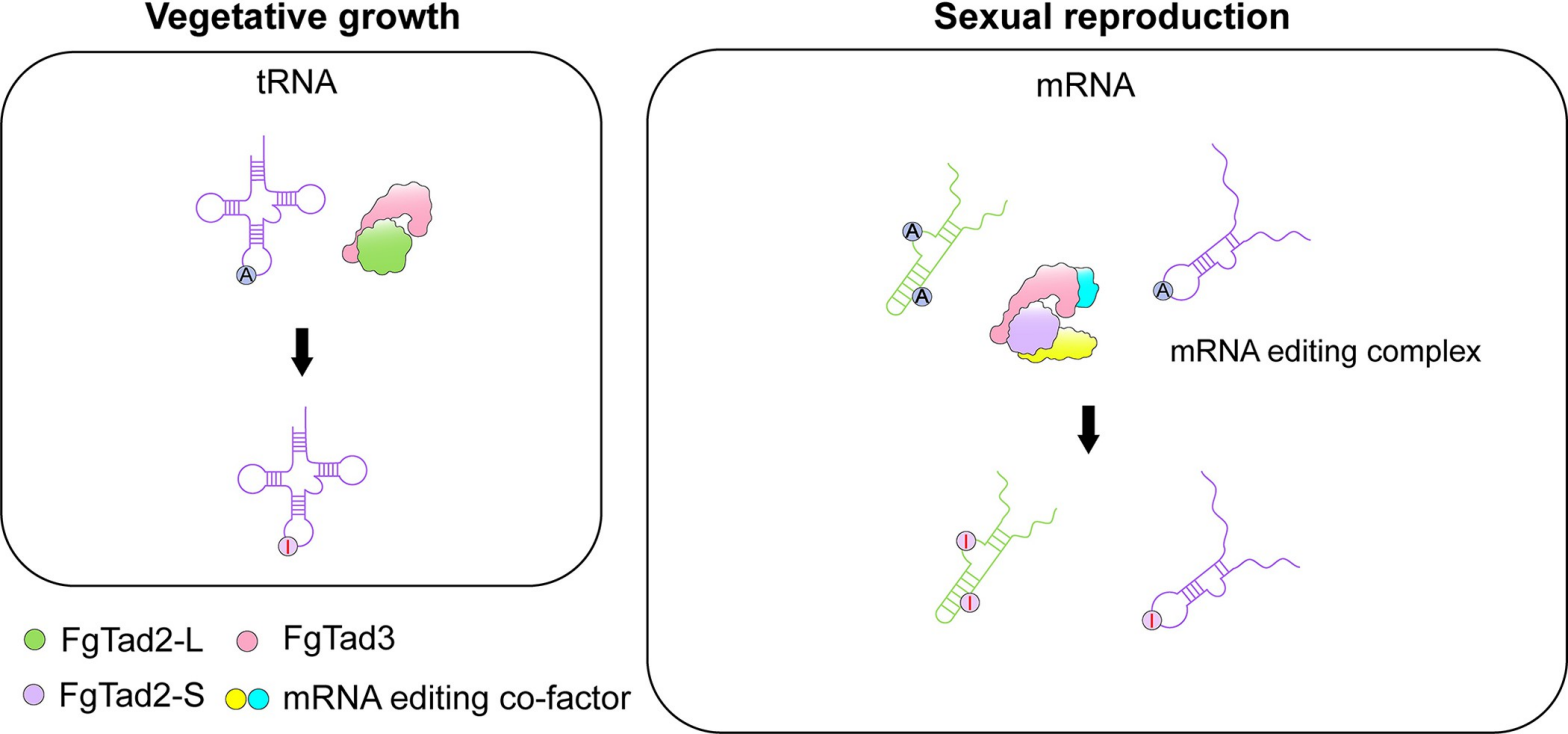
A model for editing of A34 in tRNA and A-to-I mRNA editing by FgTad2 and FgTad3. In vegetative hyphae, the FgTad2L and FgTad3 heterodimer functions as regular ADAT to edit A34 in the anticodon loop of tRNA. During sexual reproduction, the expression of short isoforms and stage-specific cofactors, including Ame1 and Fip5, enable the editing of adenosines in the hair loops (favored) and stems (dsRNA regions) of mRNA by the FgTad2S-FgTad3-cofactor protein complex.

FgTad3, like yeast Tad3, is enzymatically inactive due to the E to V mutation in the catalytic core but its forms heterodimers with FgTad2 [6,23]. The same RIP approach was used to identify mutations in *FgTAD3*. Among the RIP mutations identified in *FgTAD3*, the M120I mutation was verified to have no effect on vegetative growth but affect perithecium development and ascospore formation. Only 40 A-to-I RNA editing sites were identified in 7-dpf perithecia formed by the *FgTAD3*^M120I^ mutants [22]. These results further indicate that the FgTad2-FgTad3 heterodimers mediate A-to-I mRNA editing in fungi (Fig 2).

## Stage-specific cofactor of ADATs for mRNA editing

Proteomics analysis with affinity-purified FgTad2-His proteins from vegetative hyphae and perithecia identified 17 proteins that are specifically associated with FgTad2 in perithecia. Some of these FgTad2-ineracting proteins (FIPs) may function as stage-specific cofactors that facilitate editing of mRNA by the FgTad2-FgTad3 ADAT heterodimers during sexual reproduction. FIP5 has an ssDNA-binding domain that contains 2 RNA recognition motifs (RRMs). Deletion of *FIP5* that is specifically expressed during sexual reproduction had no effect on vegetative growth. However, the *fip5* mutant produced only small perithecia with aborted asci lack of ascospores. In RNA isolated from 6-dpf perithecia, no A-to-I editing could be detected at A1831 and A1834 of *PUK1* and A655 of *RTT1* in the *fip5* mutant. However, editing still occurred at A1358 of *RTT2*, indicating that *FIP5* is not essential but may play a faciliatory role in RNA editing during sexual reproduction [23].

The *AME1* (activator of mRNA editing 1) gene was identified by functional characterization of genes that are specifically expressed in perithecia and conserved between *F*. *graminearum* and *N*. *crassa* [22]. The *ame1* deletion mutant was blocked in early stages of perithecium development and RNA editing. Interestingly, overexpression of *AME1* lead to the detection of hundreds of editing events in vegetative hyphae. In yeast two-hybrid assays, Ame1 directly interacts with the N-terminal region of FgTad3. In heterogenous systems coexpressing *AME1* with the short isoforms of *FgTAD2* and *FgTAD3*, limited numbers of RNA editing events could be detected in the budding yeast and *Escherichia coli* as well as HEK 293T cells [22], confirming the importance of Ame1 in RNA editing. However, it is noteworthy here that rare *AME1* transcripts are present in vegetative hyphae and Ame1 was one of the proteins copurified with FgTad2-His in all 3 replicates of perithecia as well as 1 replicate of vegetative hyphae [23], suggesting that *AME1* is not specifically expressed during sexual reproduction in *F*. *graminearum*. *AME1* appears to evolve rapidly and its ortholog from *Sclerotinia sclerotiorum* is not functional in *F*. *graminearum* [22], but RNA editing has been observed and verified in sexual fruiting bodies of *P*. *confluens*, which is a Pezizomyces.

## Summary remarks

Based on studies in *F*. *graminearum*, *N*. *crassa*, and *S*. *macrospora*, genome-wide A-to-I mRNA editing likely occurs in many other Sordariomycetes during sexual reproduction. However, it remains to be determined which fungal groups outside Sordariomycetes other than some Pezizomycetes species have sexual specific genome-wide RNA editing. It is also not clear whether A-to-I RNA editing occurs in other developmental or infection stages (such as sclerotium formation) in filamentous fungi. Editing of effector mRNAs, if occurs during infectious growth, will enable fungal pathogens to increase the complexity of effectors without increasing the number of effector genes. Furthermore, although rare, TadA ADAT of *Escherichia coli* can catalyze A-to-I mRNA editing without the involvement of other factors [25]. Because ADATs are highly conserved, rare A-to-I RNA editing events catalyzed by Tad2/Tad3 orthologs may also occur in vegetative hyphae without stage-specific cofactors or shorter isoforms.

Regarding stage-specific cofactors, although Ame1 and Fip5 have been shown for their involvement in RNA editing in *F*. *graminearum*, they are only 2 of the FgTad2-interacting proteins identified by affinity purification and proteomics analysis [23]. It is likely that other FgTad2- or FgTad3-interacting proteins also are involved in various aspects of A-to-I RNA editing in fungi. Unlike editing of A34 that occurs specifically in the anticodon loop of tRNA, approximately one-third of the A-to-I RNA editing sites are in the predicted stem (dsRNA) regions of fungal mRNA. Therefore, Fip5 and other proteins may be important for facilitating the FgTad2-FgTad3 heterodimer to bind with dsRNA regions for editing. In addition, both *F*. *graminearum* and *N*. *crassa* have editing sites that are unique to specific development stages from 3 dpf to 8 dpf, which may involve some of these FgTad2- or FgTad3-interacting proteins in a developmental stage-specific manner. Furthermore, it is not clear what factors are responsible for recognizing specific sequences for editing and determining the editing levels at specific editing sites. Systematic identification and characterization of FgTad2- and FgTad3-interacting proteins may lead to a comprehensive understanding of the A-to-I RNA editing machinery in fungi, which will be beneficial to improve our understanding of ADAR-mediated RNA editing in metazoans.
